# Bimetallic Niobium-Based Catalysts Supported on SBA-15 for Hydrodeoxygenation of Anisole

**DOI:** 10.1021/acs.iecr.1c02799

**Published:** 2021-12-17

**Authors:** Daniel Ballesteros-Plata, Isabel Barroso-Martín, Juan Andrés Medina Cervantes, Carmen Maciel, Rafael Huirache-Acuña, Enrique Rodríguez-Castellón, Antonia Infantes-Molina

**Affiliations:** † Departamento de Química Inorgánica, Cristalografía y Mineralogía (Unidad Asociada al ICP-CSIC), Facultad de Ciencias, Universidad de Málaga, Campus de Teatinos, 29071 Málaga, Spain; ‡ Facultad de Ingeniería Química, Universidad Michoacana de San Nicolás de Hidalgo, Ciudad Universitaria, C.P. 58060, Morelia, Michoacán, México

## Abstract

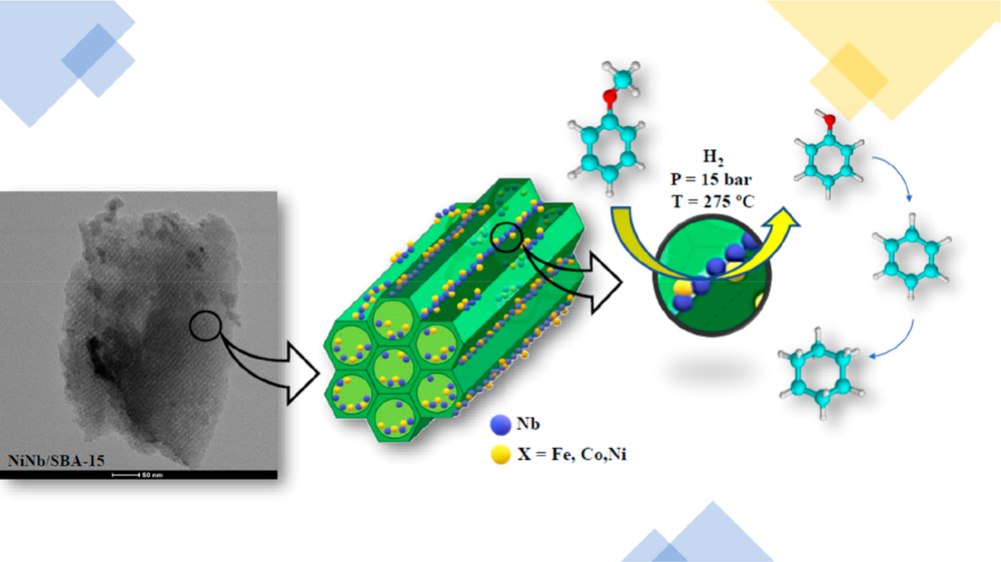

The effect of adding iron, cobalt or nickel to a prepared niobium-supported catalyst using mesoporous silica SBA-15 as a support was evaluated in the hydrodeoxygenation (HDO) reaction of anisole, chosen as a model compound in lignocellulosic biomass derived bio-oil. HDO activity as well as selectivity toward O-free products were highly dependent on the catalyst formulation: Ni incorporation showed the highest anisole conversion and selectivity to deoxygenated products, followed by Co and Fe counterparts. The activity was explained in terms of acidity, metal surface exposure and reducibility as a function of the interaction between the phases present. Regarding the characterization results, the better performance of NiNb/SBA-15 was associated with its lower acidity, higher Nb/Si surface exposure, NbO_2_/Nb_2_O_5_ ratio and better interaction between Ni and Nb species.

## 1 Introduction

The obtention of liquids fuels from biomass has gained great attention recently, since the scientific community is looking for cleaner energy solutions that not only supply the increasing energetic demand, but also that will be friendly with the environment.^1−4^ The production of transportation bifuels from biomass can be conducted by different synthetic routes: gasification to produce syngas, hydrothermal liquefaction or fast pyrolysis to produce bio-oil.^5,6^ The bio-oil includes many oxygenated hydrocarbons (alcohols, aldehydes, carboxylic acids, ketones, and phenolics), that confer undesired properties, such as thermal instability, polymerization, low calorific value and storage difficulties, as well as immiscibility with fossil-fuel-derived compounds. Therefore, a significant upgrading is required before it can be used as transportation fuel.^6,7^ In this regard, the oxygen content of the bio-oil can reach up to 50%, for bio-oil from fast pyrolysis.^8−10^

Recently, one of the approaches performed to lower the oxygen content in bio-oil is hydroprocessing, being that hydrotreatment one of the most important processes in the petroleum industry, where several heteroatoms can be removed in the presence of hydrogen and a proper catalyst (hydrodesulfurization (HDS), hydrodenitrogenation (HDN), and hydrodeoxygenation (HDO) to remove S, N, and O, respectively). In many occasions, these hydrotreatments are accompanied by processes of hydrogenation (saturation) of olefins and aromatic compounds.^11^ The upgrading of bio-oil through HDO partially or totally removes the oxygenated compound, being the most common upgrading route, despite its high consumption of hydrogen and the requirement of high pressures at moderate temperatures (300–600 °C).^9,12^ A possible solution that can allow a reduction in hydrogen pressure is the preparation of effective bifunctional HDO catalysts, which combine supports with acidic nature^13−15^ and metal centers with HDO activity as niobium-containing catalysts. Niobium-based catalysts are becoming promising materials capable of catalyzing reactions such as oxidation, hydration, dehydration, hydrolysis, esterification, hydrodeoxygenation, alkylation, condensation, and photocatalysis, since they are acid, stable and present high tolerance to water.^16,17^ Dumesic et al.^18^ were the first to report a niobium-containing catalyst, Pt/NbOPO_4_, for total HDO reaction. Later, Wang et al.^19^ observed that Pt/NbOPO_4_ was more active than Pt/SiO_2_–Al_2_O_3_ in the reaction of ring-opening/hydrogenation of 4-(2furyl)-3buten-2-one) to octane. That catalyst, under very mild conditions (165–175 °C, 25 bar), was able to not only convert octanediols to octane via dehydration/hydrogenation but also to convert 4-(2-tetrahydrofuryl)-butan-2-ol to octane via dehydration, ring-opening and hydrogenation. In addition, a Pd catalyst supported on niobium phosphate was active in the HDO of triglycerides to obtain C7–C8 alkanes favoring the hydrogenolysis of ester groups and suppressing cleavage of the C–C bond.^20^

Over the past few years, various noble metals and niobium-based catalysts have been employed in HDO reactions with good catalytic results.^21−24^ However, despite having a high hydrogenation capacity, the main drawback of using noble metals is their scarcity and high price. Therefore, the scientific community is looking for new active phases based on non-noble transition metals to reduce costs. Among them, transition metals such as Ni, Co or Fe are known to act as promoters and can also catalyze hydrogenation.^25^ Ni atom shows an affinity for H_2_ molecules assisting hydrogenation.^26^ Nickel has been used extensively in hydrogenation, since Sabatier discovered its activity, and it is one of the most used hydrotreating catalysts, because it is active even using water as a hydrogen source.^27^ It is generally accepted that the catalytic activity of catalysts containing nickel has great dependence on the surface acidity. Pichler et al.^28^ studied the influence of the synthesis methods, in terms of elements contamination for Ni/ZrO_2_ catalyst in the HDO of guaiacol and reported that the remaining elements of the preparation of the support like Si or Na changed the surface acidity and lowers catalytic activity. Cobalt is an inexpensive active phase, compared to precious metals. It is a component of the typical CoMoS/Al_2_O_3_ hydrotreating catalyst and is active for HDO.^29^ HDO of guaiacol was performed using both Ni/ZrP and Co/ZrP catalysts by Han et al.,^30^ and they showed different reactions pathways, affecting selectivity, demonstrating that cobalt favors a less hydrogen-consuming reaction pathway, producing phenol and cyclohexane as main products. In addition, iron has been cited as a good promoter, because of its abundance, low cost, and effective catalytic performance improvement. In the gas-phase upgrading of guaiacol, Fe/SiO_2_ was chosen as a catalyst to study the ability of Fe atoms to break hydroxyl and methoxyl bonds in aromatic rings.^31^ The latter has shown oxophilic properties that favor the direct deoxygenation pathway in the HDO mechanism.^32^ Similarly, Fe nanoparticles supported on mesoporous silica nanoparticles have demonstrated great ability to obtain diesel-range hydrocarbons from raw microalgal oil.^33^

The problems derived from using a real bio-oil have led to the use of model compounds such as guaiacol, phenol and anisole to simplify the analysis and understand the reaction mechanisms and kinetics involved. These three compounds comprise a large fraction (30%–40%) of lignocellulosic biomass-derived bio-oil, and the C–O bond is difficult to break.^34,35^ Anisole hydrodeoxygenation has been studied over several bifunctional catalysts. Using noble metals, a considerable hydrogenation activity has been reported with good stability, obtaining cyclohexane as the main product.^36^ Similarly, the use of supported nickel catalysts has demonstrated a good hydrogenation capability at high temperature and pressure.^37^ Also, the addition of promoters like zinc or gallium to nickel-based catalysts has shown an enhancement of the selectivity to aromatics.^38^

Therefore, the present study aimed to evaluate how incorporating a second metal into a niobium-supported catalyst can influence the textural, structural, acidic and catalytic properties of the resulting XNb/SBA-15 catalysts (with X = Fe, Co, or Ni). As far as we are concerned, the addition of Fe, Co or Ni to Nb supported on SBA-15 catalysts has not been studied yet in this reaction. The resulting bifunctional catalysts have been fully characterized before testing in the HDO reaction of anisole. Anisol has been chosen because of its intermediate complexity between guaiacol (two separated functional groups) and phenol besides its small size, which makes it highly resistant to deoxygenation, so that the HDO of anisole on these catalysts should ensure the deoxygenation of other reactive molecules, such as phenol.

## 2 Experimental Section

### 2.1 Reagents

SBA-15 support was synthesized using the following reagents: poly(ethylene glycol)-*block*-poly(propylene glycol)-*block*-poly(ethylene glycol) (Pluronic P123, Sigma–Aldrich); sulfuric acid (95%, Technical VWR Prolabo Chemicals); sodium hydroxide (Technical VWR Prolabo Chemicals) and sodium silicate solution (25%–28%, Sigma–Aldrich).

Iron(III) nitrate nonahydrate (Fe(NO_3_)_3_·9H_2_O, Sigma–Aldrich); Cobalt(II) nitrate hexahydrate (Co(NO_3_)_2_·6H_2_O, Sigma–Aldrich); Nickel(II) nitrate hexahydrate (Ni(NO_3_)_2_·6H_2_O, Alfa Aesar) and niobium(V) oxalate hydrate (C_10_H_5_NbO_20_·H_2_O, Alfa Aesar) were used as precursor salts for bimetallic catalysts synthesis. Oxalic acid dihydrate (C_2_H_2_O_4_·2H_2_O, Scharlau Chemie) was used to dissolve niobium(V) oxalate hydrate.

### 2.2 Catalysts Synthesis

The synthesis method described by Cazalilla et al.^39^ was followed to obtain a low-cost SBA-15 mesoporous support. Three bimetallic catalysts were prepared by incipient wetness impregnation, containing 12 wt % of metal (X+Nb) loading, with X = Fe, Co or Ni and an X/Nb molar ratio equal to 1 in all cases, resulting in catalysts containing a Nb loading of 7.7, 7.3 and 7.3 wt % for Fe-, Co- and Ni-based catalysts, respectively. In a typical synthesis, the SBA-15 support was first impregnated with niobium oxalate solution in oxalic acid 0.1 M. After impregnation, Nb/SBA-15 precursor was dried for 24 °C at 60 °C and then calcined at 450 °C for 2 h. The corresponding Fe, Co and Ni saline solutions (4.6 wt % experimentally) were then impregnated into the Nb/SBA-15 precursor. The catalytic precursors (FeNb/SBA-15, CoNb/SBA-15 and NiNb/SBA-15) were dried and calcined following the same procedure as with Nb/SBA-15. The catalysts were respectively named FeNb, CoNb and NiNb.

### 2.3 Catalytic Activity

The catalysts were tested in HDO of anisole as a model compound present in lignocellulosic biomass-derived bio-oil. The reaction was performed in a high-pressure, continuous down-flow fixed-bed reactor. The temperature was fixed at 275 °C and controlled with a thermocouple placed inside the stainless-steel reactor in close contact with the catalytic bed consisting of 250 mg of pelletized catalyst (particle size = 0.85–1 mm) diluted with SiC up to 3 cm^3^. The feed containing 2 wt % anisole in *cis*–*trans* decahydronaphtalene was supplied with a Gilson 307S piston pump. The catalysts were reduced in situ at 450 °C for 2 h, using a hydrogen flow of 100 mL min^–1^ and a heating rate of 10 °C min^–1^. After cooling, the reactions were performed at 275 °C and 15 bar of H_2_ with a liquid feed flow of 0.18 mL min^1^ and a hydrogen flow of 30 mL min^1^ with a liquid hourly space velocity of 3.6 h^–1^ and a gas hourly space velocity of 600 h^–1^, and hydrogen contact time of 6 s.

HDO conversion was calculated using eq 1:
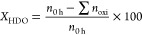
1where *n*_0 h_ is the initial amount of anisole and *n*_oxi_ is the amount of oxygenated compounds.

Every 60 min, liquid samples were collected and analyzed by gas chromatography, using a Shimadzu Model GC-14B system that was equipped with a TBR-14 capillary column.

### 2.4 Characterization of Samples

Powder X-ray diffraction (PXRD) high-angle measurements (10°–70° in 2*θ*, 0.0167° step size) were performed on a PAN analytical X’Pert Pro automated diffractometer in Bragg–Brentano reflection configuration, using a Ge(111) primary monochromator, with monochromatic Cu Kα radiation (λ = 1.5406 Å) and an X′Celerator detector. Low angle patterns (1°–5° in 2*θ*) were collected in a BRUKER D8 Discover diffractometer, with a Gobel mirror with a 0.3° point slit and a collimator of the same diameter in the primary beam. The detector was a two-dimensional EIGER system from DECTRIS.

To know the textural properties of the support and catalysts, nitrogen adsorption–desorption isotherms at −196 °C were performed on a Micromeritics ASAP 2020 apparatus. Before the analysis, the samples were outgassed at 150 °C for 10 h.

TALOS Model F200x equipment working both in high-resolution transmission electron microscopy (HRTEM) and STEM modes was used for the obtention of HRTEM images in order to analyze morphology and particle size distribution. Microanalysis were performed using a EDX Super-X system with four X-ray detectors and an X-FEG beam.

In order to study the reducibility of the samples, temperature-programmed reduction in flowing H_2_ (H_2_-TPR) analyses were performed. Prior to the analysis, the sample was cleaned in flowing helium (35 mL min^–1^) at 100 °C for 30 min. The sample was cooled in helium afterward up to 45 °C and then heated up to 700 °C with a heating rate of 10 °C min^–1^, using a hydrogen flow of 45 mL min^–1^, registering the signal with a Shimadzu Model GC-14B gas chromatograph equipped with a thermal conductivity detection (TCD) device.

The strength and concentration of acid centers for the reduced catalysts were determined by temperature-programmed desorption of ammonia (NH_3_-TPD). First, 80 mg of reduced catalyst were placed in a quartz sample holder and cleaned using flowing helium (35 mL min^1^) and heating from room temperature to 550 °C. Then, the sample was cooled in helium up to 100 °C, and NH_3_ was passed at this temperature for 5 min. Physisorbed ammonia was removed by cleaning the surface with flowing helium for a half hour and, lastly, the sample was heated up to 550 °C with a heating rate of 10 °C min^–1^, using helium (35 mL min^1^) as a gas carrier for registering the signal using a Shimadzu Model GC-14B gas chromatograph that was equipped with a TCD detector.

The surface chemical composition of the reduced catalysts was evaluated by X-ray photoelectron spectra (XPS) measurements in order to evaluate surface chemical composition of the catalysts. To this end, a Physical Electronics Model PHI 5701 spectrometer that used nonmonochromatic Mg K radiation (300 W, 15 kV, 1253.6 eV) and was equipped with a multichannel detector. Experiments were performed in a constant pass energy mode at 29.35 eV and Si 2*p* (103.4 eV) was used for charge referencing.

## 3 Results and Discussion

### 3.1 Catalytic Test

**Figure 1:**
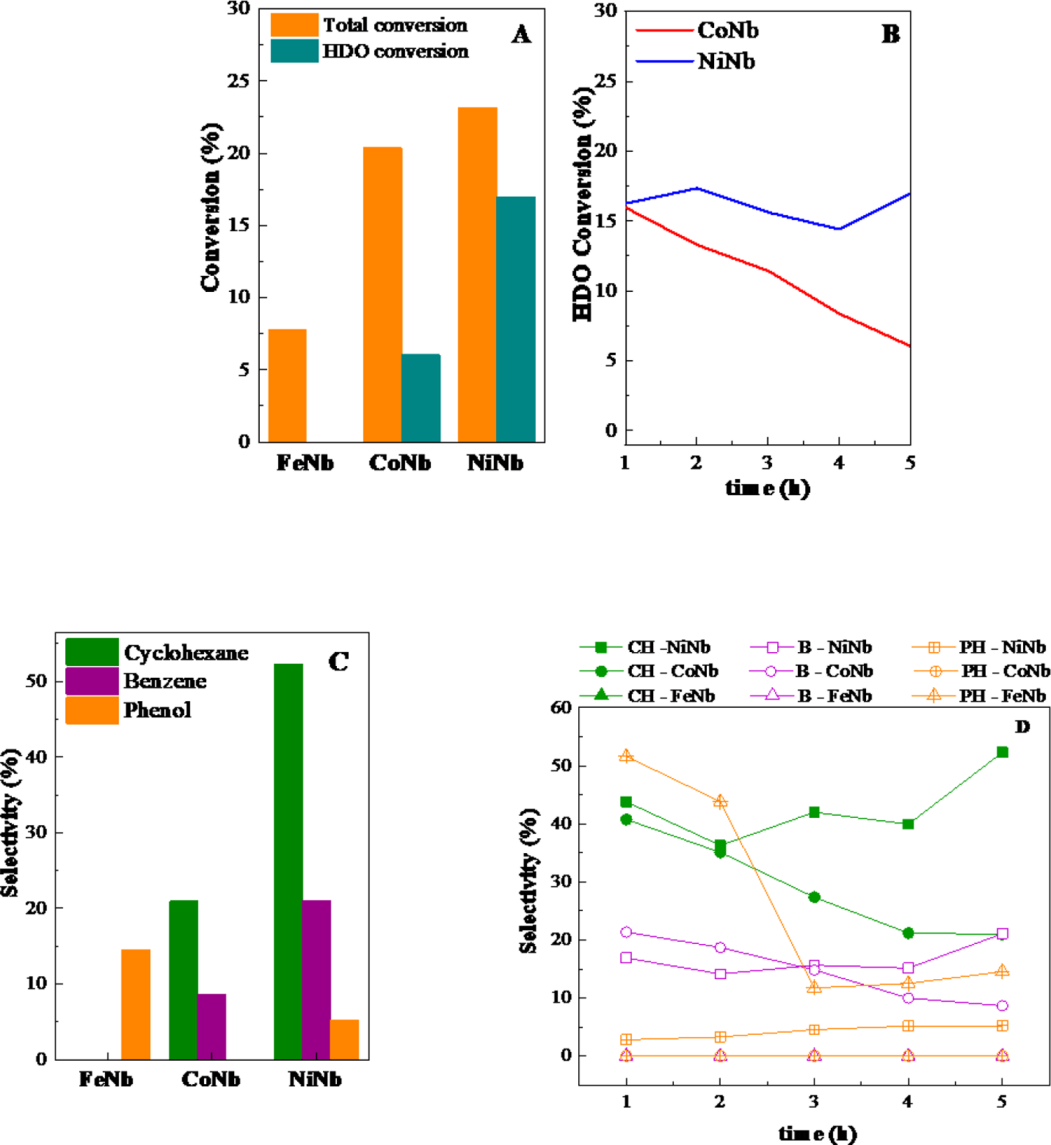
(A) Conversion of anisole after 5 h on stream; (B) evolution of HDO conversion of anisole with time on stream; (C) selectivity data for the studied catalysts after 5 h; and (D) selectivity evolution with time on stream.

The catalytic activity of Nb-based catalysts was studied in a continuous down-flow fixed-bed reactor at 15 bar and 275 °C, using anisole as a model molecule. The reaction was monitorized for 5 h, and the results are compiled in Figure 1. Both total and HDO conversions after 5 h on stream are depicted in Figure 1A, where it is evidenced significant differences in the conversion capacity depending on the catalyst used. Thus, the HDO conversion corresponding to FeNb catalyst was observed to be zero throughout the entire reaction time and, in addition, the total conversion attained was lower than 10%. This fact could be ascribed to a weaker interaction between Nb and Fe due to the latter larger ionic radius. In fact, taking into account the position of these metals in the periodic table, it can be observed that the ionic radius follows the trend Fe > Co > Ni, which is analogous to the HDO activity trend FeNb < CoNb < NiNb, suggesting that ions with a smaller radius would interact more easily with the Nb species. All this is also reflected in the product distribution, where the NiNb catalyst gave rise to a high selectivity towards deoxygenated products, contrary to that observed with CoNb and especially with FeNb. Even though in 5 h of reaction it is not possible to speak of catalytic stability, note that the NiNb catalyst seems to show a more stable behavior throughout the 5 h of reaction (Figure 1B). In contrast, the CoNb catalyst is deactivated as the reaction proceeds. The analysis of spent samples evidenced that the amount of coke followed the trend: FeNb (7.6%) > CoNb (6.7%) > NiNb (4.1%) which could explain the observed deactivation of the samples.

**Scheme 1:**
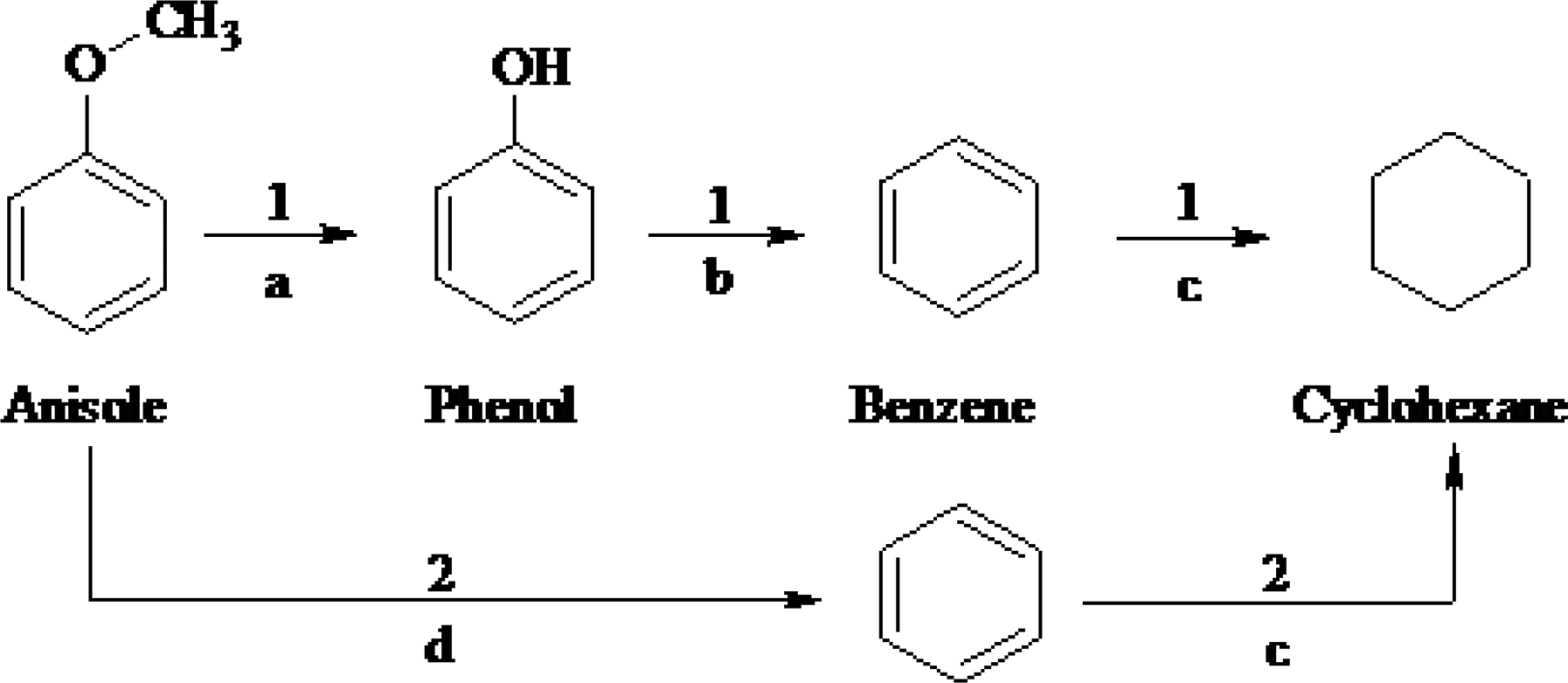
Possible Reaction Pathways for HDO Reaction for Anisole

Considering the detected product selectivity (Figure 1C), the main reaction products detected were cyclohexane and benzene for the CoNb and NiNb catalysts and phenol for the FeNb catalyst. In no case the formation of molecules such as *o*-cresol, toluene, orthoxylene, cyclohexanol or methoxycyclohexane was observed, as reported by others.^40−42^ In order to justify the proposed reaction pathway, the evolution of detected products with time has been included in Figure 1D, from where it is suggested that the most likely reaction mechanism is the one shown in Scheme 1, where there are two main routes:

- *Route 1:* the demethylation (DM) of the O–CH_3_ bond occurs, giving rise to phenol (step a). The direct hydrogenolysis of phenol (HDO) led to the formation of benzene (step b).^43^ Finally, the hydrogenation of the saturated benzene ring occurs to obtain cyclohexane (step c).^29^
- *Route 2:* simultaneously, the demethylation and the hydrogenolysis of anisole molecule occur to directly obtain benzene (step d), which, as occurs in route 1, can be hydrogenated to cyclohexane (step c).^40^

According to studies by Xia et al.^44^ and Shao et al.,^45^ Nb species have a significant promotional effect for C–O bond cleavage. This fact will be corroborated in the H_2_-TPR study. The incorporation of Ni resulted in the best distribution of HDO products with the highest percentage of deoxygenated products (cyclohexane and benzene), and negligible amounts of oxygenated intermediates were obtained, close to 5%, which indicates that it probably followed route 1 of the proposed reaction mechanism (Scheme 1). In contrast, the Co-based catalyst only gave rise to benzene and cyclohexane (route 2 of Scheme 1), without observing oxygenated intermediates at any time during the reaction. Lastly, the FeNb catalyst did not deoxygenate the anisole molecule under the reaction conditions studied. Looking at literature data, it is reported that, although the metal sites play a key role in H_2_ activation, hydrogenation of the aromatic ring of anisole probably takes place in the acid sites of the supports. Thus, it is reported that during the anisole conversion on SBA-based catalysts, the anisole molecule can interact with silanol (−OH) groups, followed by hydrogenation of the aromatic ring on the metal sites.^40^

### 3.2 Characterization Results

#### 3.2.1 X-ray Diffraction

**Figure 2:**
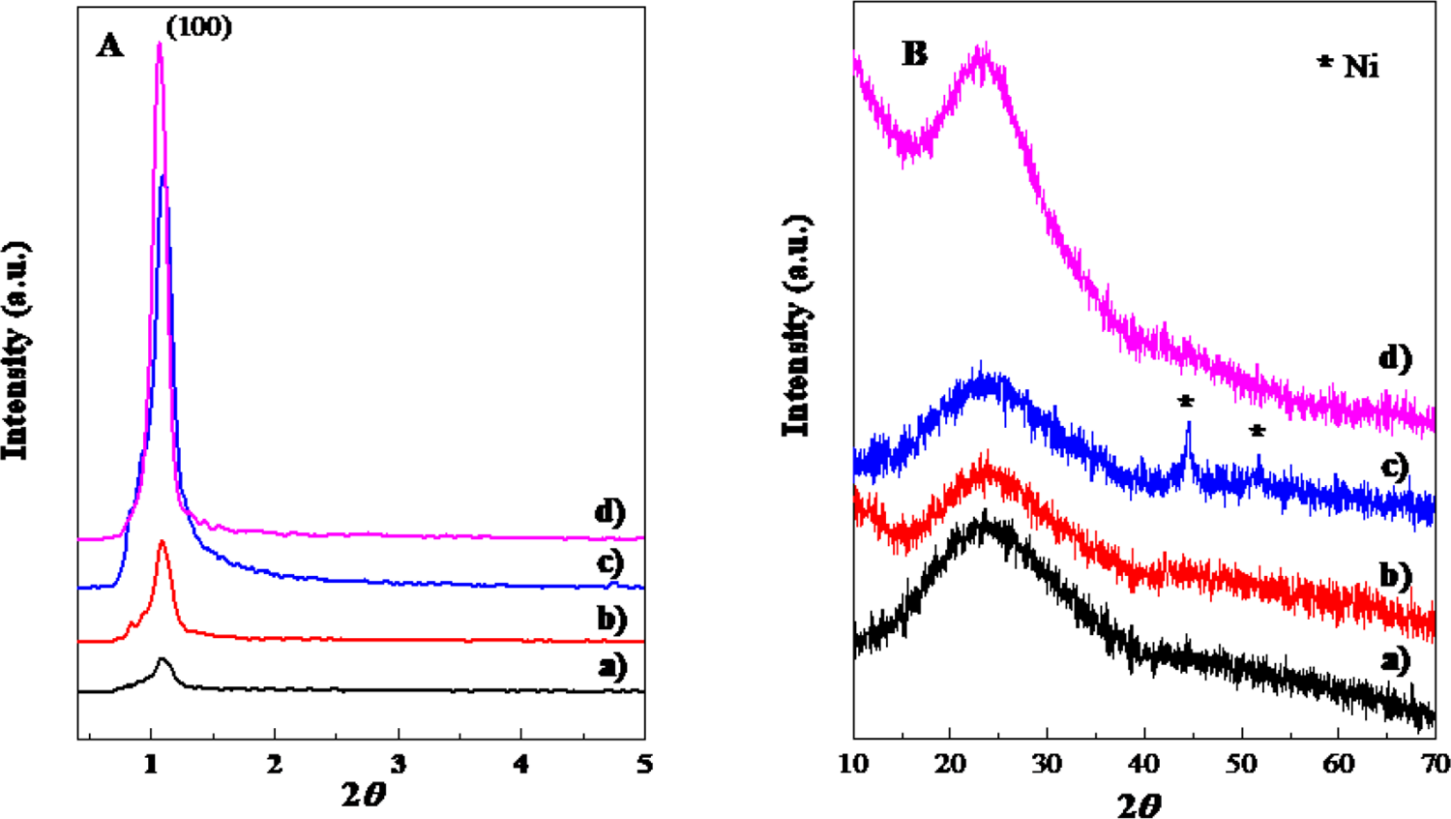
XRD patterns at (A) low and (B) high angles for (a) FeNb, (b) CoNb and (c) NiNb reduced catalysts, and (d) the SBA-15 support.

XRD measurements at low angle were performed to elucidate the degree of ordering in the structure of the as-prepared catalysts. For the bare support (Figure 2A), an intense peak at 2*θ* = 1° shows that SBA-15 preserves the hexagonal order of the silica.^46−48^ When the metallic phases were incorporated, a decrease in the intensity of the peak was observed at 1°, especially with cobalt and iron. This fact indicates that a greater disorder in the SBA-15 porous structure is occurring after the addition of cobalt and iron than in the case of nickel. Generally, it is observed that the incorporation of metals affects the porous structure, producing pores blockages. According to the results obtained and supported by *S*_BET_ measurements, this effect is more pronounced in Fe and Co catalysts, because of the larger ionic radius of these elements. The same results were obtained by Kilos et al.^49^ with SBA-15 supported Nb-based catalysts.

Figure 2B shows the wide-angle XRD patterns of all reduced catalysts and bare support. A broad hump at 20°–30° was observed in the 2*θ* region, associated with the amorphous nature of mesoporous materials. The absence of any diffraction peaks corresponding to segregated Nb oxide phases in all reduced catalysts and the diffraction peaks of the Fe and Co species can be observed in the FeNb and CoNb catalysts, respectively. Nevertheless, in the case of the NiNb catalyst, two weak peaks can be matched with reflections of metallic Ni at 2*θ* = 44.5° and 51.7° (PDF No. 03-065-2865).^50^ These results indicate that the metallic phases are widely dispersed on the support. The fact that no reflections lines of Nb, Fe and Co are observed (and only two weak signals of metallic Ni) may be a consequence of the too small sizes of the particles^51^ probably located within the porous channels of the SBA-15 support, dispersed on the surface of the wall or forming small groups that are barely detected by XRD.

The presence of Nb, Fe and Co in the SBA-15 type silica was confirmed by TEM images.

#### 3.2.2 N_2_ Adsorption–Desorption Measurements

**Figure 3:**
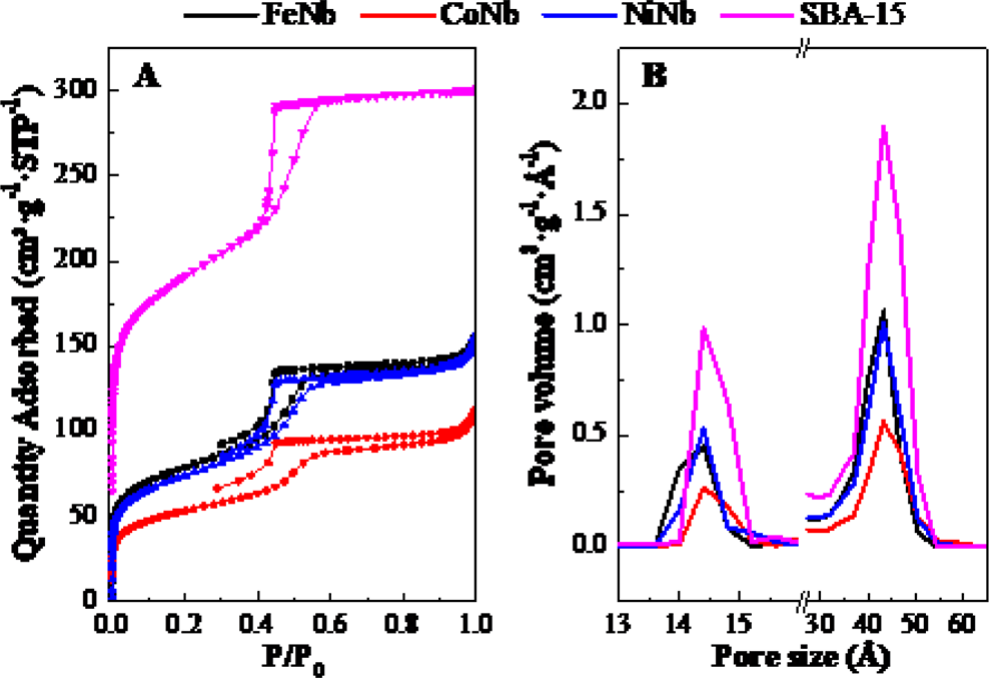
(A) N_2_ adsorption–desorption isotherms and (B) micropore and mesopore size distribution by MP and DFT method, respectively, of the supported reduced bimetallic catalysts and the bare support.

Figure 3A reports the N_2_ adsorption–desorption isotherms of the reduced catalysts and bare support. The isotherms are almost identical and are of type IV according to the IUPAC classification,^52^ characteristic of mesoporous materials. At low relative pressures, a strong increase in the volume of adsorbed N_2_ is observed, indicating that the samples contain a considerable amount of micropores. Therefore, it could be said that the sample is micro-mesoporous, which is characteristic of the SBA-15 support, where mesopores are interconnected to each other by micropores.^53,54^ Nevertheless, because of the mean microporosity and mesoporosity values obtained when analyzing the isotherms, it is observed that the samples have quite small mesopores and large micropores (Figure 3B). As can be seen in Figure 3A, the desorption isotherms of the catalysts do not close the hysteresis cycle, because, at relative pressures close to 0.30, the nitrogen molecules that remain to be desorbed are chemisorbed inside the micropores, and the pressure is not enough to desorb the nitrogen in the very small pores and probably very sinuous, because the metallic particles are also hosted inside them. Hysteresis loops do not differ from one catalyst to another, exhibiting an H2-type shape typical of ordered mesoporous materials, in which percolation occurs due to interconnection in the pore network. At *P*/*P*_0_ > 0.45, all isotherms show a characteristic step due to capillary nitrogen condensation within the mesopores. The isotherms of the catalysts also present a narrow hysteresis loop that extends to very high relative pressures, close to 0.99, which indicates the presence of large mesopores between particles.

**Figure 4:**
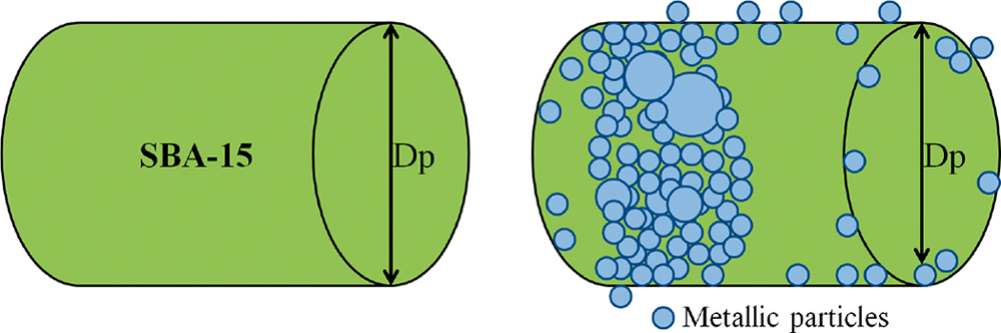
Possible mechanism of deposition of the active phase into the pores of the support.

**Figure 5:**
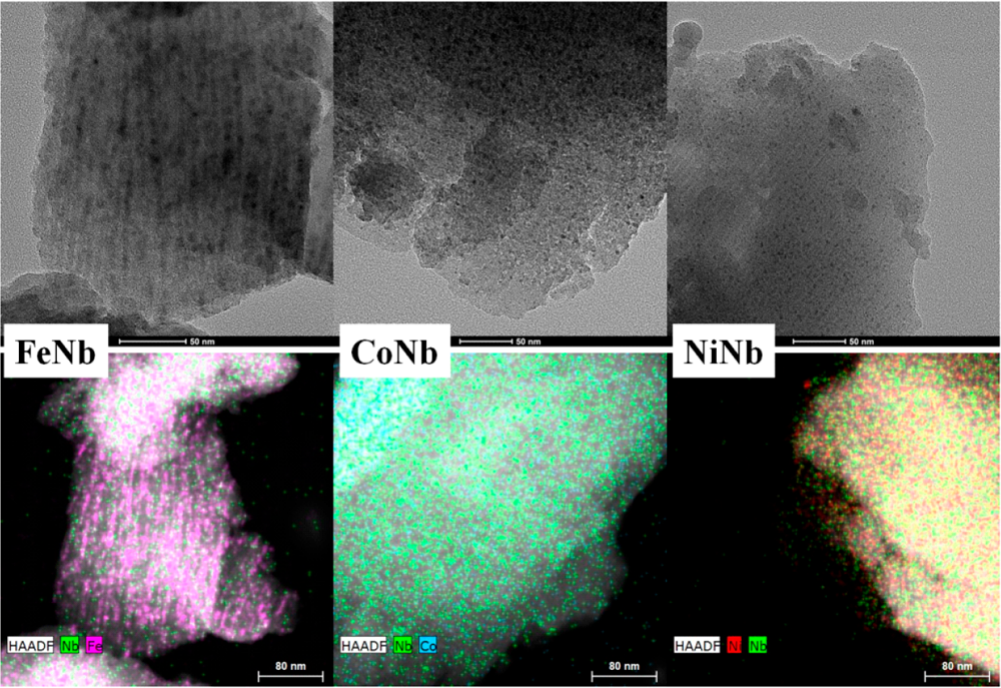
HRTEM micrographs and mapping results corresponding to the reduced catalysts.

The incorporation of the metallic phases into the SBA-15 support resulted in a great decrease both in the amount of N_2_ adsorbed and in the BET surface area, compared to the pure support. Nevertheless, the pore size distribution does not change after the deposition of metallic phases (Table 1). The decrease in the pore volume, both micropores and mesopores, could be due to the blockage of the pores by the metallic particles (see Figure 4), which is greater with cobalt, which also justifies that the pore size is the same in the three catalysts and in the bare support (Figure 3B). The particle size of the active phases is so small (see Figure 5) that it hardly produces any variation in the pore size of the catalysts, with respect to the pure support. However, it could be happening that the entrance to a certain number of pores is obstructed by larger metal particles and/or by agglomerates of small particles, preventing the entry of N_2_ and thus causing a decrease in the observed pore volume (Figure 3B). The same results were described by Hewer et al.^40^ when Ni and Mo were incorporated into the SBA-15 support. All this would also serve to justify the HDO conversion obtained by the catalysts.

**Table 1:** Summary of Textural Properties of the Support and the Catalysts

sample	*S*_BET_^a^ (m^2^ g^–1^)	*V*_meso_^b^ (cm^3^ g^–1^)	*V*_micro_^c^ (cm^3^ g^–1^)	*D*_meso_^d^ (nm)	*D*_micro_^e^ (nm)
SBA-15	693	0.50	0.081	4.34	1.41
FeNb	272	0.21	0.030	4.32	1.39
CoNb	188	0.13	0.019	4.32	1.40
NiNb	261	0.19	0.027	4.34	1.38

a *S*_BET_ = Brunauer–Emmett–Teller specific surface area.

b Volume of mesopore determined using the DFT method.

c Volume of micropore determined using the MP method.

d Average mesopore width calculated using the DFT method.

e Average micropore diameter calculated using the MP method.

#### 3.2.3 Transmission Electron Microscopy (TEM)

The distribution and size of metallic particles on the SBA-15 were investigated by means of high-resolution transmission electron microscopy (HRTEM) and energy-dispersive X-ray analysis (EDX). Figure 5 shows the corresponding micrographs and mapping analysis. In all cases, it is observed that the active phases are well-dispersed on the support and the particle sizes are very small, as was also deduced from the N_2_ adsorption and XRD analysis. In the case of FeNb catalyst, it is observed that iron particles are located inside the SBA-15 channels while niobium does not seem to interact so much with iron particles. Mapping analysis confirmed this fact, with small Nb particles (green spotspresent all over the sample Fe (purple spots) was localized inside the porous of the SBA-15. Instead, it seems that the Co–Nb and Ni–Nb interactions are better, with the metallic particles being more homogeneously distributed as can be seen in Figure 5.

#### 3.2.4 Temperature-Programmed Reduction of H_2_ (H_2_-TPR)

**Figure 6:**
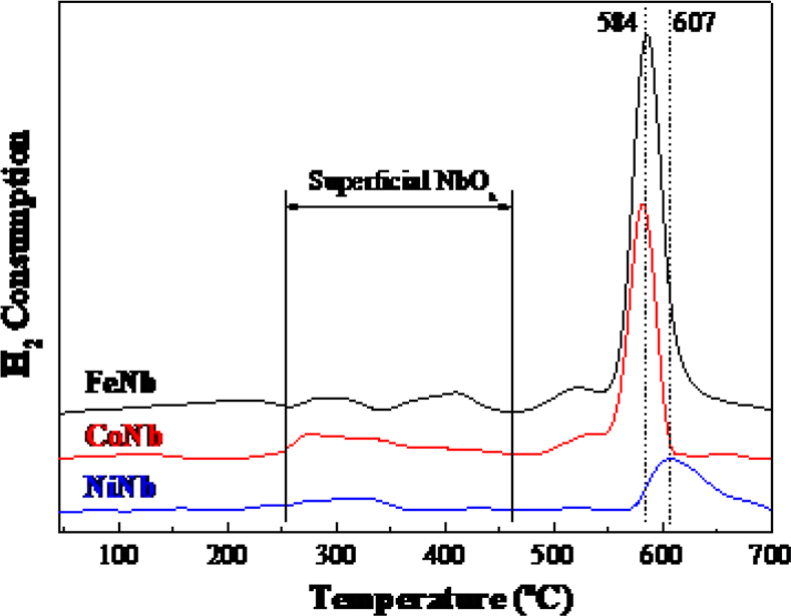
H_2_-temperature-programmed reduction profiles for bimetallic catalysts.

H_2_-TPR studies were performed for the three bimetallic oxide catalysts to determine the effect of adding a second metal on a catalyst based on niobium. The H_2_-TPR profile for each catalyst shows a main hydrogen consumption peak, narrow and located near 600 °C (Figure 6). For the FeNb/SBA-15 and CoNb/SBA-15 catalysts, this peak appears at ∼584 °C and for the NiNb/SBA-15 one, the hydrogen consumption signal shifts to slightly higher temperatures (607 °C). In any case, these three signals of hydrogen consumption are due to the presence of Nb_2_O_5_ species reducible to NbO_2_,^10,42,43^ as will be see with the XPS analysis. Regarding the reducibility of the niobium species, note that the reduction of the Nb_2_O_5_ species to NbO_2_ is reversible and therefore, the reoxidation of NbO_2_ gives rise to Nb_2_O_5_.^55^

Other weaker reduction peaks are observed between 250 and 475 °C, which are due to the reduction of superficial NbO_*x*_ species,^55^ nickel oxide,^56^ cobalt oxide^57^ or iron oxide,^58^ respectively. Similar to that observed with the main reduction peaks (close to 600 °C), it is observed that the reduction profiles in the 250–475 °C range are more intense for the FeNb sample and less intense for the NiNb one. This fact again shows that the interaction between Ni and Nb is stronger than in the case of Fe–Nb and Co–Nb pairs, as reported in previous investigations,^59,60^ since hydrogen consumption during reduction is lower, so there will be fewer species of niobium available to consume hydrogen. Just the opposite occurs with iron, which seems to weakly interact with niobium, leading to a large consumption of hydrogen, and in the middle is cobalt.

These results are in accordance with those obtained in catalysis, where NiNb catalyst showed the best conversion and selectivity toward deoxygenated products, followed by CoNb and FeNb, probably due to the greater Ni–Nb interaction detected in the H_2_-TPR study.

#### 3.2.5 Temperature-Programmed Desorption of Ammonia (TPD-NH_3_)

**Figure 7:**
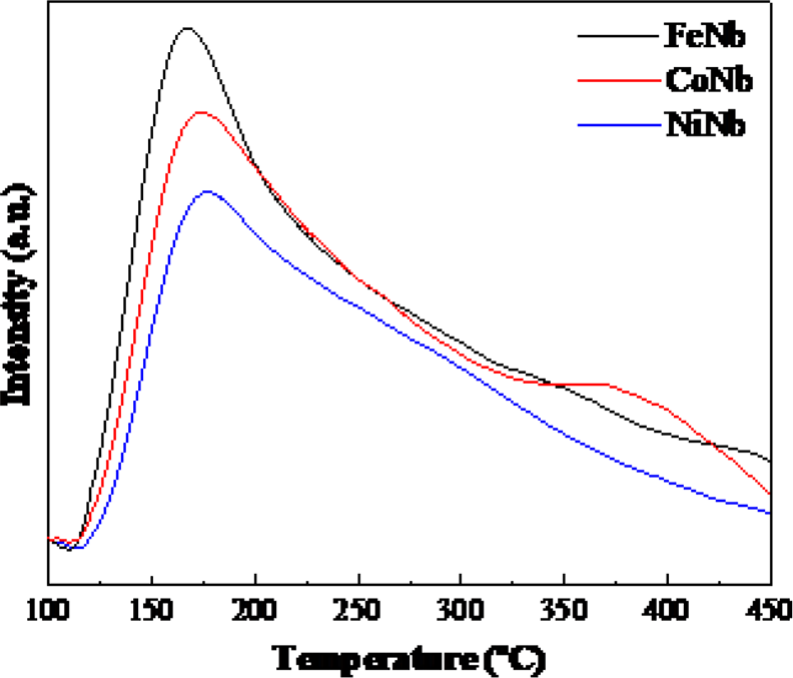
NH_3_-temperature-programmed desorption profiles for reduced bimetallic catalysts.

Acidity was analyzed from NH_3_-TPD and Figure 7 displays NH_3_-TPD curves for the reduced samples. All catalysts presented a broad desorption peak centered at 175 °C and the ammonia desorption occurred mainly at low temperature being the desorption complete at 450 °C. Table 2 shows the amount of NH_3_ desorbed (μmol NH_3_ g^–1^) for each sample. Quantification results reveal that the acidity is mainly of a weak nature for the three samples, since the main desorption occurs at low temperature, between 100 and 300 °C in the three as-prepared catalysts. This acidity is attributed to niobium oxide species, NbO_2_ (as will be seen later in XPS), where Lewis acid sites are mainly present.^40,61^ According to the study performed by Yakovlev et al.^29^ with bimetallic catalysts based on Ni and Cu supported on silica, selectivity toward benzene rings occurs mainly when active sites of weak acidity are present, which would coincide with the catalytic results obtained in this study.

**Table 2:** Acidic Properties of the Reduced Bimetallic Catalysts Determined by NH_3_-TPD

	Acidity (μmol NH_3_ g^–1^)		
catalyst	weak^a^	medium^b^	total
FeNb	56.7	11.5	68.1
CoNb	51.4	15.4	66.8
NiNb	44.9	10.7	55.6

a NH_3_ desorbed between 100 °C and 300 °C.

b NH_3_ desorbed between 300 °C and 450 °C.

In the temperature range of 300–450 °C, the catalysts present a low medium acidity associated with the niobium oxide species Nb_2_O_5_ (see XPS analysis). Only with CoNb, a slight increase in the medium acidity is observed with respect to FeNb and NiNb. Nevertheless, the total acidity reveals the behavior shown by the catalysts in the HDO reaction of anisole. So, as the total acidity decreases, the HDO conversion of anisole increases.

#### 3.2.6 X-ray Photoelectron Spectroscopy (XPS)

**Figure 8:**
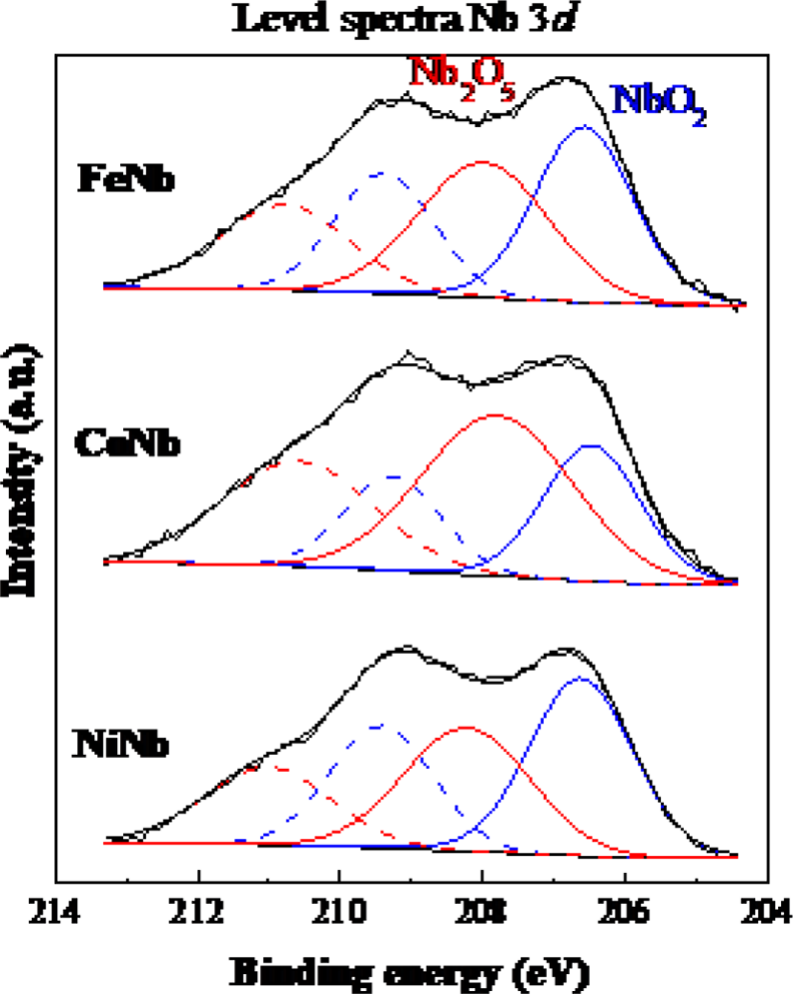
Nb 3*d* core level spectra for all studied reduced catalysts.

**Figure 9:**
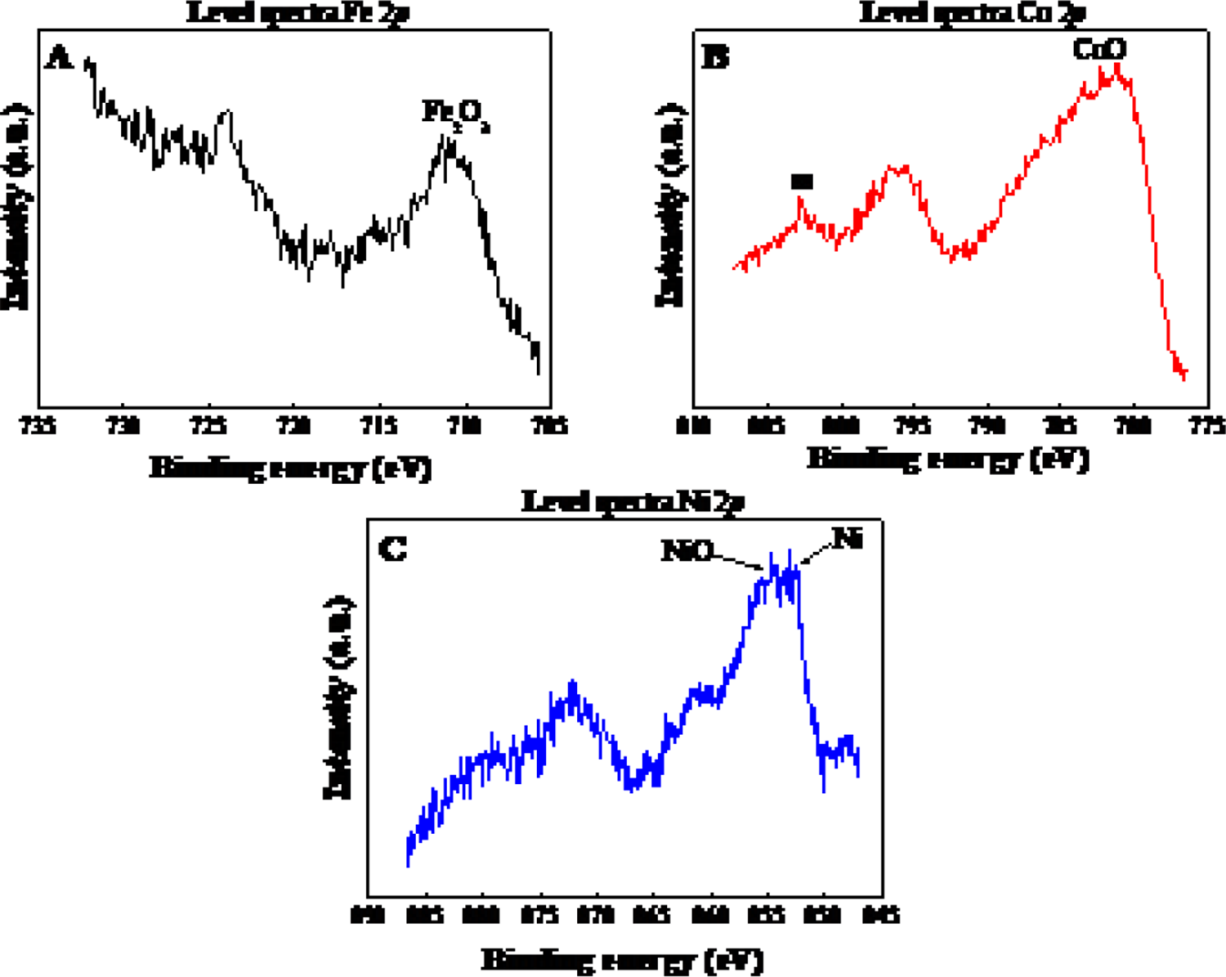
Core-level spectra of (A) Fe 2*p*, (B) Co 2*p* and (C) Ni 2*p* for samples FeNb, CoNb, and NiNb, respectively.

XPS spectra were recorded to investigate the surface chemical composition of the reduced catalysts. Table 3 includes the corresponding binding energy values of the niobium species on the surface, and Figures 8 and 9 show the spectra for the different elements studied. The surface analysis of the samples indicated that the Nb/Si atomic ratio is higher for the NiNb catalyst, which indicates that there is a greater amount of exposed niobium species on the catalyst surface. The predominant species of niobium in the catalysts are NbO_2_ and Nb_2_O_5_ (Figure 8) and the relative percentage of each oxide varies from one catalyst to another (Table 3).

**Table 3:** Binding Energy Values for Nb 3*d*_5/2_ and Surface Composition

	Binding Energy^a^ (eV)				
	Nb 3*d*_5/2_		Surface Composition		Binding Energy (eV)
catalyst	NbO_2_	Nb_2_O_5_	NbO_2_/Nb_2_O_5_	Nb/Si	M_*x*_O_*y*_
FeNb	206.6 (51)	208.0 (49)	1.02	0.026	710.8^b^ (Fe_2_O_3_)
CoNb	206.5 (36)	207.8 (64)	0.57	0.026	781.2^c^ (CoO)
NiNb	206.6 (55)	208.2 (45)	1.22	0.034	852.5^d^ (Ni)
					854.3^d^ (NiO)

a Values given in parentheses indicate the relative percentage of each species.

b Fe 2*p*_3/2_.

c Co 2*p*_3/2_.

d Ni 2*p*_3/2_.

Nb and NiNb catalysts present similar compositions of both species, being the ratio (NbO_2_/Nb_2_O_5_) > 1 and slightly higher in the NiNb configuration. However, it is not the case with the CoNb catalysts, which presents higher surface concentration of Nb_2_O_5_ species than of NbO_2_ ones. According to the acidity results obtained by NH_3_-TPD, the higher concentration of acid centers of a medium nature obtained for the CoNb catalyst would be due to the presence of Nb_2_O_5_ species, and the low acidity should be mainly caused by the NbO_2_ species. The spectra of the reduced catalysts in Figure 9 show the surface presence of oxidized species of Fe, Co and Ni (oxides of Nb are also observed in Figure 8). Only the metallic phase of Ni^0^ is observed in the NiNb catalyst, which corresponds to the results obtained in the XRD analysis.

## 4 Conclusions

The effect of incorporating Fe, Co and Ni to a low-cost SBA-15 supported Nb-based catalyst has been evaluated in the anisole hydrodeoxygenation (HDO) reaction performed at 275 °C and a hydrogen pressure of 15 bar. The results obtained showed that there is a stronger interaction between nickel and niobium than between Co–Nb and Fe–Nb, which led to a better conversion of HDO and a greater selectivity toward deoxygenated molecules. The greatest interaction force shown between Ni and Nb was verified by the temperature-programmed reduction of H_2_, where the Nb_2_O_5_ species were reduced to NbO_2_ in a lower number and with greater difficulty (at ∼600 °C), compared to what was observed in the FeNb and CoNb catalysts. However, by XPS the NiNb catalyst was shown to have a higher surface percentage of NbO_2_ species than Nb_2_O_5_ species. This fact contrasts with that observed by H_2_-TPR; however, it must be taken into account that the catalysts used during the reaction were reduced to 450 °C for 2 h prior to the reaction. According to the results consulted in the bibliography and those obtained in this study, in this temperature range, surface NbO_*x*_ species are mainly reduced, which confirms the highest percentage of NbO_2_ species present on the catalytic surface observed through XPS analysis, which gives rise to a higher Nb/Si ratio than in the FeNb and CoNb catalysts. Furthermore, the NbO_2_ species are responsible for the low acidity of a weak nature shown by these catalysts, especially by the NiNb catalyst, which favors the HDO process.

## References

[ref1] Bykova M. V.; Ermakov D. Y.; Kaichev V. V.; Bulavchenko O. A.; Saraev A. A.; Lebedev M. Y.; Yakovlev V. Ni-Based Sol-Gel Catalysts as Promising Systems for Crude Bio-Oil Upgrading: Guaiacol Hydrodeoxygenation Study. Appl. Catal., B 2012, 113–114, 296–307. 10.1016/j.apcatb.2011.11.051.

[ref2] Wang W.; Yang Y.; Luo H.; Hu T.; Liu W. Amorphous Co-Mo-B Catalyst with High Activity for the Hydrodeoxygenation of Bio-Oil. Catal. Commun. 2011, 12 (6), 436–440. 10.1016/j.catcom.2010.11.001.

[ref3] Kumar M.; Olajire Oyedun A.; Kumar A. A Review on the Current Status of Various Hydrothermal Technologies on Biomass Feedstock. Renewable Sustainable Energy Rev. 2018, 81, 1742–1770. 10.1016/j.rser.2017.05.270.

[ref4] Oyedun A. O.; Kumar A.; Oestreich D.; Arnold U.; Sauer J. The Development of the Production Cost of Oxymethylene Ethers as Diesel Additives from Biomass. Biofuels, Bioprod. Biorefin. 2018, 12 (4), 694–710. 10.1002/bbb.1887.

[ref5] Huber G. W.; Iborra S.; Corma A. Synthesis of Transportation Fuels from Biomass: Chemistry, Catalysts, and Engineering. Chem. Rev. 2006, 106, 4044–4098. 10.1021/cr068360d. 16967928

[ref6] Bridgwater A. V. Review of Fast Pyrolysis of Biomass and Product Upgrading. Biomass Bioenergy 2012, 38, 68–94. 10.1016/j.biombioe.2011.01.048.

[ref7] Mullen C. A.; Boateng A. A. Chemical Composition of Bio-Oils Produced by Fast Pyrolysis of Two Energy Crops. Energy Fuels 2008, 22 (3), 2104–2109. 10.1021/ef700776w.

[ref8] Patel M.; Oyedun A. O.; Kumar A.; Gupta R. A Techno-Economic Assessment of Renewable Diesel and Gasoline Production from Aspen Hardwood. Waste Biomass Valorization 2019, 10 (10), 2745–2760. 10.1007/s12649-018-0359-x.

[ref9] Furimsky E. Catalytic Hydrodeoxygenation. Appl. Catal., A 2000, 199, 147–190. 10.1016/S0926-860X(99)00555-4.

[ref10] Maggi R.; Delmon B. Characterization and Upgrading of Bio-Oils Produced by Rapid Thermal Processing. Biomass Bioenergy 1994, 7 (1–6), 245–249. 10.1016/0961-9534(94)00062-X.

[ref11] Wang H.; Male J.; Wang Y. Recent Advances in Hydrotreating of Pyrolysis Bio-Oil and Its Oxygen-Containing Model Compounds. ACS Catal. 2013, 3 (5), 1047–1070. 10.1021/cs400069z.

[ref12] Sanna A.; Vispute T. P.; Huber G. W. Hydrodeoxygenation of the Aqueous Fraction of Bio-Oil with Ru/C and Pt/C Catalysts. Appl. Catal., B 2015, 165, 446–456. 10.1016/j.apcatb.2014.10.013.

[ref13] Yang Y.; Ochoa-Hernandez C.; de la Pena O'Shea V. A.; Pizarro P.; Coronado J. M.; Serrano D. P. Effect of Metal-Support Interaction on the Selective Hydrodeoxygenation of Anisole to Aromatics over Ni-Based Catalysts. Appl. Catal., B 2014, 145, 91–100. 10.1016/j.apcatb.2013.03.038.

[ref14] Yan P.; Kennedy E.; Stockenhuber M. Natural Zeolite Supported Ni Catalysts for Hydrodeoxygenation of Anisole. Green Chem. 2021, 23 (13), 4673–4684. 10.1039/D0GC04377J.

[ref15] Tu C.; Chen J.; Li W.; Wang H.; Deng K.; Vinokurov V. A.; Huang W. Hydrodeoxygenation of Bio-Derived Anisole to Cyclohexane over Bi-Functional IM-5 Zeolite Supported Ni Catalysts. Sustain. Energy Fuels 2019, 3 (12), 3462–3472. 10.1039/C9SE00554D.

[ref16] Nowak I.; Ziolek M. Niobium Compounds: Preparation, Characterization, and Application in Heterogeneous Catalysis. Chem. Rev. 1999, 99 (12), 3603–3624. 10.1021/cr9800208. 11849031

[ref17] Tanabe K. Catalytic Application of Niobium Compounds. Catal. Today 2003, 78 (1–4), 65–77. 10.1016/S0920-5861(02)00343-7.

[ref18] West R. M.; Liu Z. Y.; Peter M.; Dumesic J. A. Liquid Alkanes with Targeted Molecular Weights from Biomass-Derived Carbohydrates. ChemSusChem 2008, 1 (5), 417–424. 10.1002/cssc.200800001. 18702136

[ref19] Xu W.; Xia Q.; Zhang Y.; Guo Y.; Wang Y.; Lu G. Effective Production of Octane from Biomass Derivatives under Mild Conditions. ChemSusChem 2011, 4 (12), 1758–1761. 10.1002/cssc.201100361. 22045599

[ref20] Xia Q.; Zhuang X.; Li M. M. J.; Peng Y. K.; Liu G.; Wu T. S.; Soo Y. L.; Gong X. Q.; Wang Y.; Tsang S. C. E. Cooperative Catalysis for the Direct Hydrodeoxygenation of Vegetable Oils into Diesel Range Alkanes over Pd/NbOPO_4_. Chem. Commun. 2016, 52 (29), 5160–5163. 10.1039/C5CC10419J. 26998532

[ref21] Guan W.; Chen X.; Zhang J.; Hu H.; Liang C. Catalytic Transfer Hydrogenolysis of Lignin α-O-4 Model Compound 4-(Benzyloxy)Phenol and Lignin over Pt/HNbWO_6_/CNTs Catalyst. *Renew*. Renewable Energy 2020, 156, 249–259. 10.1016/j.renene.2020.04.078.

[ref22] Guan W.; Chen X.; Hu H.; Tsang C. W.; Zhang J.; Lin C. S. K.; Liang C. Catalytic Hydrogenolysis of Lignin β-O-4 Aryl Ether Compound and Lignin to Aromatics over Rh/Nb_2_O_5_ under Low H2 Pressure. Fuel Process. Technol. 2020, 203, 106392 10.1016/j.fuproc.2020.106392.

[ref23] Guan W.; Chen X.; Li C.; Zhang J.; Tsang C. W.; Hu H.; Li S.; Liang C. Nb(Ta)-Based Solid Acid Modified Pt/CNTs Catalysts for Hydrodeoxygenation of Lignin-Derived Compounds. Mol. Catal. 2019, 467, 61–69. 10.1016/j.mcat.2019.01.015.

[ref24] Guan W.; Chen X.; Jin S.; Li C.; Tsang C. W.; Liang C. Highly Stable Nb_2_O_5_-Al_2_O_3_ Composites Supported Pt Catalysts for Hydrodeoxygenation of Diphenyl Ether. Ind. Eng. Chem. Res. 2017, 56 (47), 14034–14042. 10.1021/acs.iecr.7b03736.

[ref25] Emmett P. H.; Skau N. The Catalytic Hydrogenation of Benzene over Metal Catalysts. J. Am. Chem. Soc. 1943, 65 (6), 1029–1035. 10.1021/ja01246a010.

[ref26] Badoga S.; Ganesan A.; Dalai A. K.; Chand S. Effect of Synthesis Technique on the Activity of CoNiMo Tri-Metallic Catalyst for Hydrotreating of Heavy Gas Oil. Catal. Today 2017, 291, 160–171. 10.1016/j.cattod.2017.01.005.

[ref27] Jin W.; Pastor-Pérez L.; Villora-Picó J. J.; Pastor-Blas M. M.; Odriozola J. A.; Sepúlveda-Escribano A.; Reina T. R. In-Situ HDO of Guaiacol over Nitrogen-Doped Activated Carbon Supported Nickel Nanoparticles. Appl. Catal., A 2021, 620, 118033 10.1016/j.apcata.2021.118033.

[ref28] Pichler C. M.; Gu D.; Joshi H.; Schüth F. Influence of Preparation Method and Doping of Zirconium Oxide onto the Material Characteristics and Catalytic Activity for the HDO Reaction in Nickel on Zirconium Oxide Catalysts. J. Catal. 2018, 365, 367–375. 10.1016/j.jcat.2018.07.021.

[ref29] Yakovlev V. A.; Khromova S. A.; Sherstyuk O. V.; Dundich V. O.; Ermakov D. Y.; Novopashina V. M.; Lebedev M. Y.; Bulavchenko O.; Parmon V. N. Development of New Catalytic Systems for Upgraded Bio-Fuels Production from Bio-Crude-Oil and Biodiesel. Catal. Today 2009, 144 (3–4), 362–366. 10.1016/j.cattod.2009.03.002.

[ref30] Han G. H.; Lee M. W.; Park S.; Kim H. J.; Ahn J. P.; Seo M. gi; Lee K. Y. Revealing the Factors Determining the Selectivity of Guaiacol HDO Reaction Pathways Using ZrP-Supported Co and Ni Catalysts. J. Catal. 2019, 377, 343–357. 10.1016/j.jcat.2019.07.034.

[ref31] Olcese R. N.; Francois J.; Bettahar M. M.; Petitjean D.; Dufour A. Hydrodeoxygenation of Guaiacol, A Surrogate of Lignin Pyrolysis Vapors, Over Iron Based Catalysts: Kinetics and Modeling of the Lignin to Aromatics Integrated Process. Energy Fuels 2013, 27 (2), 975–984. 10.1021/ef301971a.

[ref32] Rodríguez-Aguado E.; Infantes-Molina A.; Ballesteros-Plata D.; Marco J. F.; Moretti E.; Finocchio E.; Cecilia J. A.; Rodríguez-Castellón E. Iron Phosphides Presenting Different Stoichiometry as Nanocatalysts in the HDO of Phenol. Catal. Today 2020, 349, 117–127. 10.1016/j.cattod.2018.05.023.

[ref33] Kandel K.; Anderegg J. W.; Nelson N. C.; Chaudhary U.; Slowing I. I. Supported Iron Nanoparticles for the Hydrodeoxygenation of Microalgal Oil to Green Diesel. J. Catal. 2014, 314, 142–148. 10.1016/j.jcat.2014.04.009.

[ref34] Foster A. J.; Do P. T. M.; Lobo R. F. The Synergy of the Support Acid Function and the Metal Function in the Catalytic Hydrodeoxygenation of *m*-Cresol. In Topics in Catalysis, Vol. 55; Springer, 2012; pp 118–128, 10.1007/s11244-012-9781-7.

[ref35] Massoth F. E.; Politzer P.; Concha M. C.; Murray J. S.; Jakowski J.; Simons J. Catalytic Hydrodeoxygenation of Methyl-Substituted Phenols: Correlations of Kinetic Parameters with Molecular Properties. J. Phys. Chem. B 2006, 110 (29), 14283–14291. 10.1021/jp057332g. 16854134

[ref36] Phan D. P.; Le V. N.; Kim J.; Lee E. Y. Controlled Hydrodeoxygenation of Lignin-Derived Anisole over Supported Pt on UiO-66 Based-Catalysts through Defect Engineering Approach. Fuel Process. Technol. 2021, 224, 107001 10.1016/j.fuproc.2021.107001.

[ref37] Li W.; Li F.; Wang H.; Liao M.; Li P.; Zheng J.; Tu C.; Li R. Hierarchical Mesoporous ZSM-5 Supported Nickel Catalyst for the Catalytic Hydrodeoxygenation of Anisole to Cyclohexane. Mol. Catal. 2020, 480, 110642 10.1016/j.mcat.2019.110642.

[ref38] Li F.-X.; Wang X.-F.; Zheng Y.; Chen J.-X. Influence of Metallic Promoters on the Performance of Ni/SiO_2_ Catalyst in the Hydrodeoxygenation of Anisole. J. Fuel Chem. Technol. 2018, 46, 75–83. 10.1016/S1872-5813(18)30005-7.

[ref39] Gómez-Cazalilla M.; Mérida-Robles J. M.; Gurbani A.; Rodríguez-Castellón E.; Jiménez-López A. Characterization and Acidic Properties of Al-SBA-15 Materials Prepared by Post-Synthesis Alumination of a Low-Cost Ordered Mesoporous Silica. J. Solid State Chem. 2007, 180 (3), 1130–1140. 10.1016/j.jssc.2006.12.038.

[ref40] Hewer T. L. R.; Souza A. G. F.; Roseno K. T. C.; Moreira P. F.; Bonfim R.; Alves R. M. B.; Schmal M. Influence of Acid Sites on the Hydrodeoxygenation of Anisole with Metal Supported on SBA-15 and SAPO-11. Renewable Energy 2018, 119, 615–624. 10.1016/j.renene.2017.12.044.

[ref41] Gamliel D. P.; Baillie B. P.; Augustine E.; Hall J.; Bollas G. M.; Valla J. A. Nickel Impregnated Mesoporous USY Zeolites for Hydrodeoxygenation of Anisole. Microporous Mesoporous Mater. 2018, 261, 18–28. 10.1016/j.micromeso.2017.10.027.

[ref42] Khromova S. A.; Smirnov A. A.; Bulavchenko O. A.; Saraev A. A.; Kaichev V. V.; Reshetnikov S. I.; Yakovlev V. A. Anisole Hydrodeoxygenation over Ni-Cu Bimetallic Catalysts : The Effect of Ni/Cu Ratio on Selectivity. Appl. Catal., A 2014, 470, 261–270. 10.1016/j.apcata.2013.10.046.

[ref43] Loricera C. V.; Pawelec B.; Infantes-molina A.; Álvarez-galván M. C.; Huirache-Acuña R.; Nava R.; Fierro J. L. G. Hydrogenolysis of Anisole over Mesoporous Sulfided CoMoW/SBA-15 (16) Catalysts. Catal. Today 2011, 172, 103–110. 10.1016/j.cattod.2011.02.037.

[ref44] Xia Q.; Cuan Q.; Liu X.; Gong X.; Lu G.; Wang Y. Angew. Chem., Int. Ed. 2014, 53, 9755–9760. 10.1002/anie.201403440. 25045056

[ref45] Shao Y.; Xia Q.; Liu X.; Lu G.; Wang Y. ChemSusChem 2015, 8, 1761–1767. 10.1002/cssc.201500053. 25876904

[ref46] Garg S.; Soni K.; Ajeeth Prabhu T.; Rama Rao K.S.; Murali Dhar G. Effect of Ordered Mesoporous ZrSBA-15 Support on Catalytic Functionalities of Hydrotreating Catalysts 2. Variation of Molybdenum and Promoter Loadings. Catal. Today 2016, 261, 128–136. 10.1016/j.cattod.2015.08.051.

[ref47] Zhu J.; Yang J.; Miao R.; Yao Z.; Zhuang X.; Feng X. Nitrogen Enriched, Ordered Mesoporous Carbons for Potential Electrochemical Energy Storage. J. Mater. Chem. A 2016, 4 (6), 2286–2292. 10.1039/C5TA09073C.

[ref48] Han J.; Zhang L.; Zhao B.; Qin L.; Wang Y.; Xing F. The N-Doped Activated Carbon Derived from Sugarcane Bagasse for CO_2_ Adsorption. Ind. Crops Prod. 2019, 128, 290–297. 10.1016/j.indcrop.2018.11.028.

[ref49] Kilos B.; Nowak I.; Ziolek M.; Tuel A.; Volta J. C. Transition Metal Containing (Nb, V, Mo) SBA-15 Molecular Sieves. Synthesis, Characteristic and Catalytic Activity in Gas and Liquid Phase Oxidation. Stud. Surf. Sci. Catal. 2005, 158, 1461–1468. 10.1016/S0167-2991(05)80498-7.

[ref50] Leal G. F.; Lima S.; Graca I.; Carrer H.; Barrett D. H.; Teixeira-Neto E.; Curvelo A. A. S.; Rodella C. B.; Rinaldi R. Design of Nickel Supported on Water-Tolerant Nb2O5 Catalysts for the Hydrotreating of Lignin Streams Obtained from Lignin-First Biorefining. iScience 2019, 15, 467–488. 10.1016/j.isci.2019.05.007. 31125909 PMC6532020

[ref51] Feliczak-guzik A.; Szczyglewska P.; Nowak I. The Effect of Metal (Nb, Ru, Pd, Pt) Supported on SBA-16 on the Hydrodeoxygenation Reaction of Phenol. Catal. Today 2019, 325, 61–67. 10.1016/j.cattod.2018.06.046.

[ref52] Sing K. S. W. Reporting Physisorption Data for Gas/Solid Systems with Special Reference to the Determination of Surface Area and Porosity. Pure Appl. Chem. 1985, 57 (4), 603–619. 10.1351/pac198557040603.

[ref53] Zhao D.; Huo Q.; Feng J.; Chmelka B. F.; Stucky G. D. Nonionic Triblock and Star Diblock Copolymer and Oligomeric Sufactant Syntheses of Highly Ordered, Hydrothermally Stable, Mesoporous Silica Structures. J. Am. Chem. Soc. 1998, 120 (24), 6024–6036. 10.1021/ja974025i.

[ref54] Zhao D.; Feng J.; Huo Q.; Melosh N.; Fredrickson G. H.; Chmelka B. F.; Stucky G. D. Triblock Copolymer Syntheses of Mesoporous Silica with Periodic 50 to 300 Angstrom Pores. Science 1998, 279, 548–552. 10.1126/science.279.5350.548. 9438845

[ref55] Wojcieszak R.; Jasik A.; Monteverdi S.; Ziolek M.; Bettahar M. M. J. Mol. Catal. A: Chem. 2006, 256, 225–233. 10.1016/j.molcata.2006.04.053.

[ref56] Tieuli S.; Mäki-Arvela P.; Peurla M.; Eränen K.; Wärnå J.; Cruciani G.; Menegazzo F.; Murzin D. Y.; Signoretto M. Hydrodeoxygenation of Isoeugenol over Ni-SBA-15: Kinetics and Modelling. Appl. Catal., A 2019, 580, 1–10. 10.1016/j.apcata.2019.04.028.

[ref57] Todorova S.; Blin J. L.; Naydenov A.; Lebeau B.; Kolev H.; Gaudin P.; Dotzeva A.; Velinova R.; Filkova D.; Ivanova I.; Vidal L.; Michelin L.; Josien L.; Tenchev K. Co3O4-MnOx Oxides Supported on SBA-15 for CO and VOCs Oxidation. Catal. Today 2020, 357, 602–612. 10.1016/j.cattod.2019.05.018.

[ref58] Benzaquén T. B.; Barrera D. A.; Carraro P. M.; Sapag K.; Alfano O. M.; Eimer G. A. Nanostructured Catalysts Applied to Degrade Atrazine in Aqueous Phase by Heterogeneous Photo-Fenton Process. Environ. Sci. Pollut. Res. 2019, 26 (5), 4192–4201. 10.1007/s11356-018-2348-9. 29860698

[ref59] Ko E. I.; Hupp J. M.; Rogan F. H.; Wagner N. J. J. Catal. 1983, 84, 85–94. 10.1016/0021-9517(83)90088-X.

[ref60] Chary K. V.R.; Lakshmi K. S.; Rao P. V. R.; Rao K. S. R.; Papadaki M. Characterization and Catalytic Properties of Niobia Supported Nickel Catalysts in the Hydrodechlorination of 1, 2, 4-Trichlorobenzene. J. Mol. Catal. A: Chem. 2004, 223, 353–361. 10.1016/j.molcata.2003.09.049.

[ref61] Datka J.; Turek A. M.; Jehng J. M.; Wachs I. E. Acidic Properties of Supported Niobium Oxide Catalysts: An Infrared Spectroscopy Investigation. J. Catal. 1992, 135, 186–199. 10.1016/0021-9517(92)90279-Q.

